# Unraveling the Clinical and Molecular Landscape of Myocardial Infarction in Behçet's Syndrome: A Comprehensive Analysis

**DOI:** 10.1002/iid3.70303

**Published:** 2025-11-27

**Authors:** Li‐Yang Zhang, Chun‐Hui She, Hu Dan, Jun Zou, Jian‐Long Guan

**Affiliations:** ^1^ Department of Rheumatology and Immunology Huadong Hospital Affiliated to Fudan University Shanghai China; ^2^ Shanghai Institute of Geriatrics and Gerontology, Huadong Hospital Affiliated to Fudan University Shanghai China; ^3^ Shanghai Key Laboratory of Clinical Geriatric Medicine, Huadong Hospital Affiliated to Fudan University Shanghai China

**Keywords:** Behçet's syndrome, bioinformatics analysis, clinical manifestation, diagnostic biomarker, myocardial infarction, phenotype

## Abstract

**Objectives:**

Behçet's syndrome (BS) is a rare inflammatory disorder with life‐threatening complications like myocardial infarction (MI), driven by vasculitis and immune‐mediated thrombosis. This study unveils distinct clinical and molecular profiles in BS patients with MI, employing gene analysis and Weighted Gene Co‐expression Network Analysis (WGCNA) to pinpoint critical pathways and therapeutic targets, enhancing diagnostic precision and treatment efficacy.

**Methods:**

A total of 2358 BS patients (25 with MI, 2333 without MI) were analyzed. Clinical characteristics were compared based on the cohort. Differential gene expression analysis was performed on four microarray datasets, by using GEO datasets, and WGCNA identified key gene modules. KEGG/GO enrichment revealed disease pathways, while diagnostic models (ROC curves, nomograms) evaluated predictive accuracy.

**Results:**

BS patients with MI exhibited longer disease duration, higher smoking rates, and increased vascular and cardiac involvement. Gene analysis identified 605 BS‐ and 523 MI‐related genes, with pathways linked to immune regulation and vascular remodeling. Three genes, TLR5, IL2RB, and KLRB1, showed strong diagnostic potential (AUC > 0.8). A nomogram integrating these genes achieved high predictive accuracy (AUC = 0.876).

**Conclusions:**

This study reveals distinct clinical and molecular profiles in BS‐associated MI, emphasizing inflammatory pathways and immune dysregulation. The findings provide a foundation for early diagnosis and personalized therapeutic strategies, advancing care for BS patients at risk for MI.

## 1 Introduction

Behçet's syndrome (BS), a rare and multifaceted systemic inflammatory disorder, is characterized by a complex interplay of genetic predisposition, immune dysregulation, and environmental factors, leading to a wide array of clinical manifestations [1]. As a form of multisystem vasculitis, BS can involve a wide array of organs, including the joints, gastrointestinal tract, central nervous system, and cardiovascular system, resulting in substantial morbidity [2]. Although the precise etiology remains incompletely understood, BS is widely regarded as a disease driven by a complex interplay of genetic predisposition, immune dysregulation, and environmental factors [3]. Recent advancements have highlighted the contribution of genetic susceptibility loci, such as HLA‐B51, to the pathogenesis of BS, along with aberrant activation of innate and adaptive immune pathways [4]. These findings underscore the central role of immune dysregulation in disease progression.

The global prevalence of BS exhibits notable geographical variability, with higher incidence rates observed in populations along the Mediterranean basin, the Middle East, and East Asia, regions historically associated with the ancient “Silk Road” [2, 5]. This distribution suggests a genetic and environmental basis for disease clustering [6]. The clinical manifestations of BS are remarkably heterogeneous, with disease severity and course varying significantly among individuals [7]. This variability, coupled with its episodic nature, often complicates diagnosis and management [8].

Although cardiovascular involvement is relatively infrequent in BS, its clinical impact is profound [7]. Myocardial infarction (MI) is among the most severe complications, posing a significant threat to patient survival [9]. Unlike traditional atherosclerotic coronary artery disease, BS‐associated MI arises from distinct pathological mechanisms, including vasculitis‐induced endothelial dysfunction, immune‐mediated thrombosis, and arterial lesions [10]. These processes result in unique clinical presentations, such as atypical chest pain, recurrent thrombosis, and exaggerated systemic inflammatory responses [11]. Such atypical manifestations often lead to delayed diagnosis and suboptimal treatment, emphasizing the need for a deeper understanding of the disease's cardiovascular pathology [12].

Advances in molecular and clinical research have increasingly highlighted the complex interplay between systemic inflammation, vascular damage, and immune dysregulation in BS‐associated MI [8]. In this context, the integration of transcriptomics and gene network analysis, holds promise for unraveling the molecular basis of this life‐threatening complication [13, 14]. Weighted Gene Co‐expression Network Analysis (WGCNA) has emerged as a powerful tool for identifying key molecular pathways and hub genes associated with disease progression, offering opportunities for the discovery of novel biomarkers and therapeutic targets [15].

**Figure 1:**
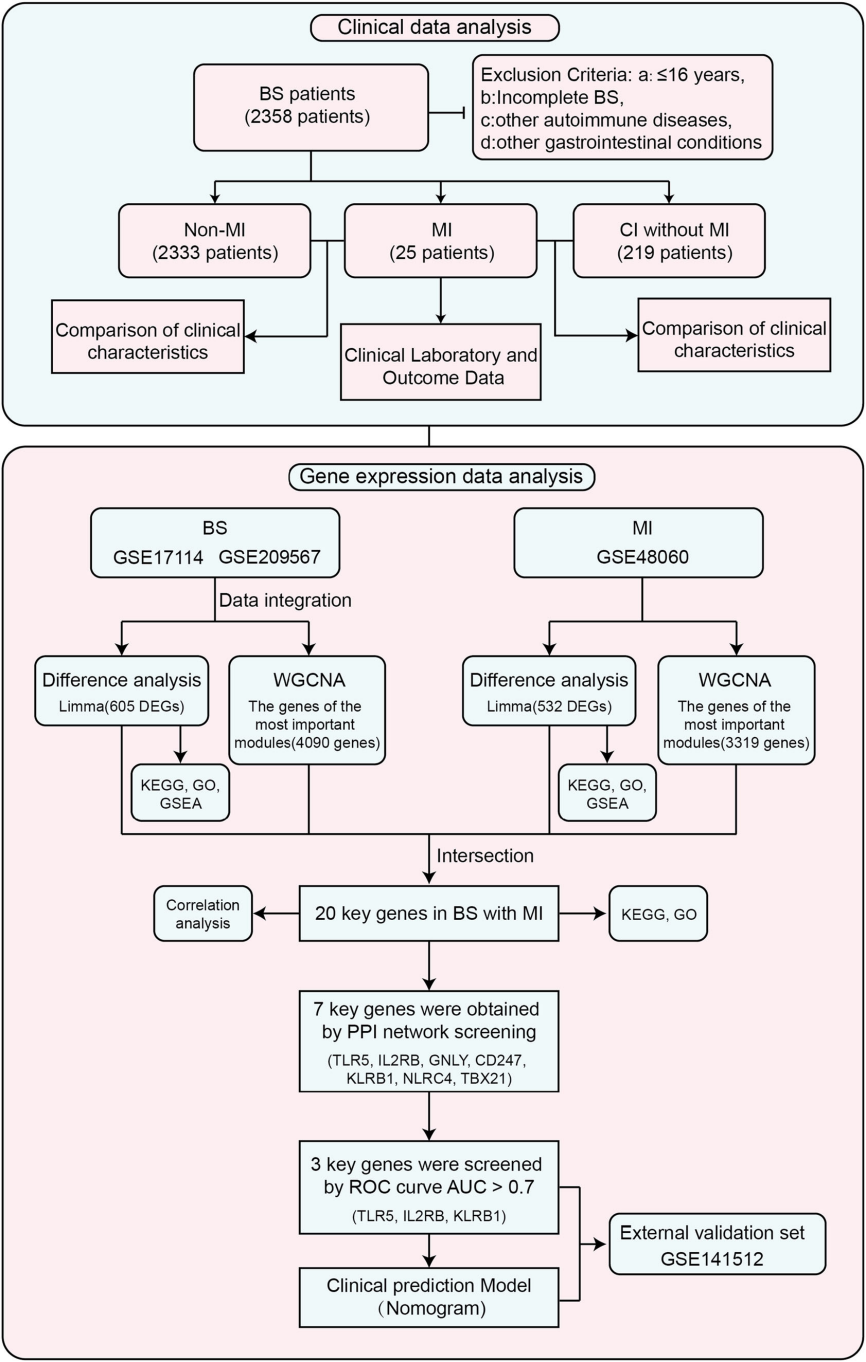
Flow chart.

This study seeks to address existing knowledge gaps by systematically characterizing the clinical features of BS patients with and without MI, while leveraging transcriptomic techniques to elucidate the molecular mechanisms underlying BS‐associated MI (Figure 1). By identifying critical gene networks and pathways implicated in immune regulation, vascular remodeling, and thrombosis, this study aims to advance diagnostic precision and pave the way for personalized therapeutic strategies. These findings have the potential to significantly enhance the early detection and management of cardiovascular complications in BS, ultimately improving patient outcomes and reducing disease burden.

## 2 Methods

### 2.1 Study Subjects

A cross‐sectional study was conducted on BS patients admitted to the Department of Rheumatology and Immunology at Huadong Hospital, affiliated with Fudan University, from September 2012 to October 2024. Patients were included based on the International Criteria for Behçet's Disease (ICBD), with exclusions for malignancies, infectious diseases, and other autoimmune conditions. BS diagnosis was based on the International Criteria for Behçet's Disease (ICBD) [16], Pediatric Behçet's Disease (PEDBD) [17], and the International Study Group (ISG) [18] criteria. Exclusion criteria included age of onset ≤ 16 years, malignancies, infectious diseases, or other autoimmune conditions. Comprehensive clinical and laboratory data were carefully reviewed, including demographic information, laboratory assessments, imaging studies, and pathological findings. This study was approved by the Ethics Committee of Huadong Hospital, and all patients provided informed consent. All methods were performed in accordance with the relevant guidelines and regulations.

### 2.2 Clinical Features and Major Organ Involvement of BS

We conducted a comprehensive analysis of patient symptoms, medical records, physical examination, laboratory tests, imaging studies, and endoscopic findings. Skin lesions included erythema nodosum, folliculitis, positive pathological examination, erythema multiforme, and thrombophlebitis [19]. Gastrointestinal manifestations of BS were confirmed by endoscopy and intestinal tuberculosis and Crohn's disease were excluded [20]. Ocular involvement includes various forms of uveitis and ocular complications [21]. Vascular involvement includes arterial thrombosis, arterial thrombosis and aneurysm [22]. Cardiac lesions were confirmed by echocardiography or coronary angiography and CT scan as endocarditis with valvular regurgitation, intracardiac thrombosis, ascending aortic aneurysm and coronary artery disease [9]. Neurological involvement includes cerebral aneurysms, cerebral thrombosis, parenchymal degeneration, and neurological disorders [23]. Patients with hematologic involvement usually present with leukopenia, thrombocytopenia, and/or anemia with/without MDS [24]. Joint system involvement often manifests as arthralgia or arthritis.

### 2.3 Data Set Acquisition

Four microarray datasets (GSE17114, GSE209567, GSE48060, and GSE141512) were downloaded from the NCBI Gene Expression Omnibus (GEO) database [25] (Supporting Information Table S1). Datasets GSE17114 and GSE209567 include gene expression data from BS patients and normal controls. Datasets GSE57691 and GSE7084 contain data from MI patients and controls.

### 2.4 Differentially Expressed Gene (DEG) and Weighted Gene Co‐Expression Network Analysis (WGCNA)

To identify DEGs and construct a weighted gene co‐expression network, we first merged the gene expression datasets GSE17114 and GSE209567. The combined data were normalized to ensure consistency across the datasets. Differential expression analysis was performed using the Limma package on the merged dataset as well as on the separate datasets GSE48060 and GSE141512 [26]. Genes were considered differentially expressed if they met the following criteria: a *p*‐value < 0.05 and a fold change (FC) > 1.2. For WGCNA, we applied a soft‐thresholding power (*β*) of 9 for BS and a *β* of 14 for MI to optimize network connectivity and ensure scale‐free topology. The adjacency matrix was then transformed into a Topological Overlap Matrix (TOM). We computed gene modules by performing hierarchical clustering based on the dissimilarity (1–TOM) between genes. Genes with similar expression patterns were grouped into distinct modules. Module detection was carried out using hierarchical clustering combined with dynamic tree cutting techniques, which enabled the identification of gene modules that exhibited significant co‐expression patterns.

### 2.5 Functional Enrichment Analysis

KEGG, GO, and GSEA analyzes were performed in R to explore the functional pathways and biological processes associated with DEGs [27]. KEGG pathway enrichment analysis was conducted using the enrichKEGG function from the clusterProfiler package, with hypergeometric testing and *p* value correction using the Benjamini‐Hochberg method (*p* < 0.05). GO enrichment analysis was also performed using the clusterProfiler package, mapping DEGs to biological processes, molecular functions, and cellular components, with significance assessed by *p* value correction (*p* < 0.05). GSEA was carried out using the fgsea package, generating a ranked gene list based on log2 fold changes and performing enrichment analysis against gene sets from MSigDB. Significance was determined through permutation‐based *p* values, with *p* < 0.05 considered significant. All results were visualized using appropriate R plotting functions.

### 2.6 Receiver Operating Characteristic Curve (ROC) and Nomogram

A *t*‐test was performed to compare the expression levels of candidate genes between the MI and control groups. ROC curves were constructed to evaluate diagnostic performance, calculating the corresponding area under the curve (AUC) and 95% confidence intervals (CI) [28]. Additionally, a nomogram was developed using R packages to convert the relative expression levels of genes into scores, which were then aggregated into a total score for predicting the incidence of BS with MI [29]. The performance of the nomogram was further validated using ROC curve analysis.

### 2.7 Statistical Analysis

Data analysis was performed using GraphPad Prism 8.0 and R programming language (version 4.2.3). Normally distributed continuous data were presented as mean ± standard deviation (x ± s) and analyzed using the two‐sample *t*‐test. Non‐normally distributed continuous data were expressed as median (range) and analyzed using the Mann‐Whitney U test. Categorical data were compared using the χ² test or Fisher's exact test, with *p* < 0.05 considered statistically significant. Two‐step cluster analysis was performed, first selecting variables as either continuous or categorical. Continuous variables included age of onset and disease duration, while categorical variables included gender, clinical features (recurrent oral ulcers, genital ulcers, skin lesions, joint involvement), and major organ involvement (ocular, gastrointestinal, cardiac, vascular, hematologic, and neurologic involvement).

## 3 Results

### 3.1 Comparison of Clinical Characteristics Between BS Patients With and Without MI

A total of 2358 BS patients were included in this study, with significant differences observed in disease duration, smoking rates, and vascular involvement between MI and non‐MI groups (Table 1). Pediatric patients aged ≤ 16 years were excluded, as were those not meeting the diagnostic criteria, including patients with incomplete BS, other autoimmune diseases (e.g., systemic lupus erythematosus, Sjogren's syndrome), or gastrointestinal conditions (e.g., Crohn's disease, intestinal tuberculosis). The clinical characteristics of the MI and non‐MI groups were compared.

**Table 1:** Comparison of clinical characteristics between BS patients with MI and BS patients without MI patients.

Variables	Total (*n* = 2358)	MI patients (*n* = 25)	Non MI patients (*n* = 2333)	*p* value
Disease course (years, mean ± SD)	10.8 (8.2)	15.1 (10.5)	10.8 (8.2)	0.037
Onset age (years, mean ± SD)	34.1 (12.0)	35.7 (11.0)	34.1 (12.0)	0.376
Smoke	272 (11.54%)	12 (48%)	260 (11.14%)	< 0.001
Drink	250 (10.6%)	10 (40%)	240 (10.29%)	< 0.001
Oral ulcerations	2265 (96.06%)	24 (96%)	2241 (96.06%)	1.000
Genital ulcerations	1794 (76.08%)	17 (68%)	1777 (76.17%)	0.474
Skin lesions	1321 (56.02%)	17 (68%)	1304 (55.89%)	0.312
Erythema nodosum	821 (34.82%)	10 (40%)	811 (34.76%)	0.737
Folliculitis	664 (28.16%)	12 (48%)	652 (27.95%)	0.046
Pathergy test positive	138 (5.85%)	6 (24%)	132 (5.66%)	< 0.001
Erythema multiform	60 (2.54%)	1 (4%)	59 (2.53%)	0.477
Thrombophlebitis	24 (1.02%)	0 (0%)	24 (1.03%)	1.000
Ocular involvement	324 (13.74%)	3 (12%)	321 (13.76%)	1.000
Panuveitis	53 (2.25%)	1 (4%)	52 (2.23%)	0.435
Anterior uveitis	36 (1.53%)	0 (0%)	36 (1.54%)	1.000
Posterior uveitis	18 (0.76%)	0 (0%)	18 (0.77%)	1.000
Uveitis of the left eye	47 (1.99%)	0 (0%)	47 (2.01%)	1.000
Uveitis of the right eye	47 (1.99%)	1 (4%)	46 (1.97%)	0.397
Bilateral uveitis	164 (6.96%)	1 (4%)	163 (6.99%)	1.000
Retinal vasculitis	32 (1.36%)	0 (0%)	32 (1.37%)	1.000
GI involvement	744 (31.55%)	6 (24%)	738 (31.63%)	0.548
Upper digestive tract ulcer	306 (12.98%)	5 (20%)	301 (12.9%)	0.453
Intestinal ulcer complications	72 (3.05%)	1 (4%)	71 (3.04%)	0.541
Vascular involvement	191 (8.1%)	10 (40%)	181 (7.76%)	< 0.001
Arterial	91 (3.86%)	10 (40%)	81 (3.47%)	< 0.001
Venous	113 (4.79%)	3 (12%)	110 (4.71%)	0.115
Cardiac involvement	219 (9.29%)	25 (100%)	194 (8.32%)	< 0.001
Aortic aneurysm	104 (4.41%)	7 (28%)	97 (4.16%)	< 0.001
Aortic regurgitation	110 (4.66%)	8 (32%)	102 (4.37%)	< 0.001
Pulmonary artery aneurysm	14 (0.59%)	0 (0%)	14 (0.6%)	1.000
Mitral regurgitation	25 (1.06%)	2 (8%)	23 (0.99%)	0.028
Tricuspid regurgitation	1 (0.04%)	1 (4%)	0 (0%)	0.011
Myocardial infarction	25 (1.06%)	25 (100%)	0 (0%)	< 0.001
Coronary stent	33 (1.4%)	15 (60%)	18 (0.77%)	< 0.001
Pericardial effusion	24 (1.02%)	3 (12%)	21 (0.9%)	0.002
Bentall procedure	41 (1.74%)	3 (12%)	38 (1.63%)	0.009
Valve replacement	24 (1.02%)	2 (8%)	22 (0.94%)	0.026
Blood involvement	144 (6.11%)	3 (12%)	141 (6.04%)	0.193
Leukocytopenia	100 (4.24%)	1 (4%)	99 (4.24%)	1.000
Thrombocytopenia	81 (3.44%)	2 (8%)	79 (3.39%)	0.211
MDS	24 (1.02%)	1 (4%)	23 (0.99%)	0.227
Trisomy 8	48 (2.04%)	1 (4%)	47 (2.01%)	0.404
CNS involvement	225 (9.54%)	2 (8%)	223 (9.56%)	1.000
Cerebral aneurysm	23 (0.98%)	0 (0%)	23 (0.99%)	1.000
Cerebral venous thrombosis	5 (0.21%)	0 (0%)	5 (0.21%)	1.000
Myelopathy	132 (5.6%)	1 (4%)	131 (5.62%)	1.000
Mental disturbance	51 (2.16%)	0 (0%)	51 (2.19%)	1.000
Joint involvement	397 (16.84%)	5 (20%)	392 (16.8%)	0.876
Arthralgia	295 (12.51%)	5 (20%)	290 (12.43%)	0.404
Arthritis	397 (16.84%)	5 (20%)	392 (16.8%)	0.876

The mean disease duration in the MI group was 15.1 ± 10.5 years, compared to 10.8 ± 8.2 years in the non‐MI group, a statistically significant difference (*p* = 0.037), indicating a longer disease course in MI patients. However, the mean age of onset did not differ significantly between the two groups, with 35.7 ± 11.0 years in the MI group and 34.1 ± 12.0 years in the non‐MI group (*p* = 0.376).

The MI group had significantly higher rates of smoking (48%) and alcohol consumption (40%) compared to the non‐MI group (11.14% and 10.29%, respectively), with both differences reaching statistical significance (*p* < 0.001). Additionally, 24% of MI patients had a positive pathergy test, significantly higher than the 5.66% observed in the non‐MI group (*p* < 0.001). Vascular involvement was also markedly more common in MI patients, particularly arterial involvement (40% vs. 7.76% in the non‐MI group, *p* < 0.001 for both overall vascular and arterial involvement).

Cardiac conditions were notably more prevalent in the MI group, with all MI patients exhibiting some form of cardiac involvement, compared to only 8.32% of non‐MI patients (*p* < 0.001). Specific cardiac conditions, including aortic aneurysm (*p* < 0.001), aortic regurgitation (*p* < 0.001), moderate mitral regurgitation (*p* = 0.028), moderate tricuspid regurgitation (*p* = 0.011), and pericardial effusion (*p* = 0.002), were significantly more common in MI patients.

Additionally, the need for coronary stents was substantially higher in the MI group (60% vs. 0.77% in the non‐MI group, *p* < 0.001). Significant differences were also observed in the frequency of Bentall procedures (*p* = 0.009) and valve replacements (*p* = 0.026), highlighting the greater cardiovascular burden experienced by MI patients.

### 3.2 Comparison of Clinical Characteristics Between BS Patients With MI and BS Patients With Cardiac Involvement but Without MI

To explore the distinct clinical profiles of BS patients MI compared to those with cardiac involvement (CI) but without MI, we conducted a comparative analysis of their characteristics. This investigation aimed to identify specific factors that differentiate these subgroups, providing insights into potential disease mechanisms and informing tailored management strategies. The study included 219 BS patients, with 25 in the MI group and 194 in the CI group, and analyzed a range of clinical parameters to assess statistically significant differences (Table 2).

**Table 2:** Comparison of clinical characteristics between BS patients with MI and BS patients with CI but without MI.

Variables	Total (*n* = 219)	BS patients with MI (*n* = 25)	BS patients with CI but without MI (*n* = 194)	*p* value
Disease course (years, mean ± SD)	11.9 (9.2)	15.1 (10.5)	11.5 (8.9)	0.071
Onset age (years, mean ± SD)	35.3 (11.8)	35.7 (11.0)	35.1 (11.9)	0.762
Smoke	69 (31.51%)	12 (48%)	57 (29.38%)	0.097
Drink	61 (27.85%)	10 (40%)	51 (26.29%)	0.229
Oral ulcerations	203 (92.69%)	24 (96%)	179 (92.27%)	1.000
Genital ulcerations	160 (73.06%)	17 (68%)	143 (73.71%)	0.714
Skin lesions	146 (66.67%)	17 (68%)	129 (66.49%)	1.000
Erythema nodosum	80 (36.53%)	10 (40%)	70 (36.08%)	0.871
Folliculitis	90 (41.1%)	12 (48%)	78 (40.21%)	0.596
Pathergy test positive	28 (12.79%)	6 (24%)	22 (11.34%)	0.143
Erythema multiform	6 (2.74%)	1 (4%)	5 (2.58%)	0.521
Thrombophlebitis	3 (1.37%)	0 (0%)	3 (1.55%)	1.000
Ocular involvement	25 (11.42%)	3 (12%)	22 (11.34%)	1.000
Panuveitis	7 (3.2%)	1 (4%)	6 (3.09%)	0.577
Anterior uveitis	3 (1.37%)	0 (0%)	3 (1.55%)	1.000
Posterior uveitis	2 (0.91%)	0 (0%)	2 (1.03%)	1.000
Uveitis of the left eye	4 (1.83%)	0 (0%)	4 (2.06%)	1.000
Uveitis of the right eye	4 (1.83%)	1 (4%)	3 (1.55%)	0.386
Bilateral uveitis	11 (5.02%)	1 (4%)	10 (5.15%)	1.000
Retinal vasculitis	3 (1.37%)	0 (0%)	3 (1.55%)	1.000
GI. involvement	44 (20.09%)	6 (24%)	38 (19.59%)	0.800
Upper digestive tract ulcer	25 (11.42%)	5 (20%)	20 (10.31%)	0.271
Intestinal ulcer complications	9 (4.11%)	1 (4%)	8 (4.12%)	1.000
Vascular involvement	69 (31.51%)	10 (40%)	59 (30.41%)	0.458
Arterial	54 (24.66%)	10 (40%)	44 (22.68%)	0.100
Venous	25 (11.42%)	3 (12%)	22 (11.34%)	1.000
Cardiac involvement	219 (100%)	25 (100%)	194 (100%)	1.000
Aortic aneurysm	104 (47.49%)	7 (28%)	97 (50%)	0.043
Aortic regurgitation	110 (50.23%)	8 (32%)	102 (52.58%)	0.085
Pulmonary artery aneurysm	14 (6.39%)	0 (0%)	14 (7.22%)	0.378
Mitral regurgitation	25 (11.42%)	2 (8%)	23 (11.86%)	0.747
Tricuspid regurgitation	1 (0.46%)	1 (4%)	0 (0%)	0.114
Myocardial infarction	25 (11.42%)	25 (100%)	0 (0%)	< 0.001
Coronary stent	33 (15.07%)	15 (60%)	18 (9.28%)	< 0.001
Pericardial effusion	24 (10.96%)	3 (12%)	21 (10.82%)	0.743
Bentall	41 (18.72%)	3 (12%)	38 (19.59%)	0.585
Valve replacement	24 (10.96%)	2 (8%)	22 (11.34%)	1.000
Blood involvement	19 (8.68%)	3 (12%)	16 (8.25%)	0.462
Leukocytopenia	12 (5.48%)	1 (4%)	11 (5.67%)	1.000
Thrombocytopenia	10 (4.57%)	2 (8%)	8 (4.12%)	0.319
MDS	4 (1.83%)	1 (4%)	3 (1.55%)	0.386
Trisomy 8	4 (1.83%)	1 (4%)	3 (1.55%)	0.386
CNS involvement	33 (15.07%)	2 (8%)	31 (15.98%)	0.385
Cerebral aneurysm	3 (1.37%)	0 (0%)	3 (1.55%)	1.000
Cerebral venous thrombosis	0 (0%)	0 (0%)	0 (0%)	1.000
Myelopathy	19 (8.68%)	1 (4%)	18 (9.28%)	0.704
Mental disturbance	3 (1.37%)	0 (0%)	3 (1.55%)	1.000
Joint involvement	34 (15.53%)	5 (20%)	29 (14.95%)	0.717
Arthralgia	33 (15.07%)	5 (20%)	28 (14.43%)	0.663
Arthritis	34 (15.53%)	5 (20%)	29 (14.95%)	0.717

The mean disease duration was slightly longer in the MI group (15.1 ± 10.5 years) than in the CI group (11.5 ± 8.9 years), but this difference did not reach statistical significance (*p* = 0.071). Similarly, the mean age of onset was comparable between the two groups (35.7 ± 11.0 years for the MI group vs. 35.1 ± 11.9 years for the CI group, *p* = 0.762).

While the MI group had a higher proportion of smokers (48%) compared to the CI group (29.38%), this difference was not statistically significant (*p* = 0.097). A similar trend was observed for alcohol consumption, with rates of 40% in the MI group and 26.29% in the CI group (*p* = 0.229).

A significant difference was observed in the prevalence of aortic aneurysms, which were less frequent in the MI group (28%) compared to the CI group (50%; *p* = 0.043). In contrast, the need for coronary stents was substantially higher in the MI group (60%) than in the CI group (9.28%; *p* < 0.001), highlighting a marked difference in the nature of cardiac interventions between the groups.

Other cardiac conditions showed varying trends. Aortic regurgitation was more common in the CI group (52.58%) than in the MI group (32%), though this difference was not statistically significant (*p* = 0.085). Moderate tricuspid regurgitation was reported in 4% of MI patients but was absent in the CI group, a difference that also did not reach significance (*p* = 0.114).

Overall, the MI group demonstrated a higher frequency of coronary interventions and a lower prevalence of aortic aneurysms compared to the CI group. These findings suggest distinct patterns of cardiac manifestations between BS patients with MI and those with cardiac involvement but without MI, offering valuable insights into the cardiovascular burden and potential pathophysiological differences in these subgroups.

### 3.3 Clinical Laboratory and Outcome Data for BS Patients With MI

We analyzed the laboratory and clinical data of BS patients with MI (Table 3). The mean hemoglobin level was 123.7 g/L, indicative of mild anemia within the cohort, while the mean platelet count was 225.2 × 10⁹/L, suggesting no significant thrombocytopenia. The mean white blood cell count was 7.716 × 10⁹/L, with a neutrophil proportion of 69.86%, reflecting a potential inflammatory response. This was further supported by elevated levels of C‐reactive protein (CRP; mean 14.41 mg/L) and an erythrocyte sedimentation rate (ESR) of 26 mm/H. These inflammatory markers, along with a mean creatinine level of 68.08 µmol/L and a uric acid level of 311.1 µmol/L, provide valuable insights into the inflammatory and metabolic state of these patients.

**Table 3:** Clinical laboratory and outcome data for BS patients with MI.

	Mean	SD	No. (%)
Laboratory examination			
Hemoglobin (g/L)	123.7	25.09	
Blood platelet (× 10^9^/L)	225.2	57.45	
White blood cell (× 10^9^/L)	7.716	2.371	
Neutrophil granulocyte (%)	69.86	9.459	
Lymphocyte (%)	20.32	8.323	
C‐reactive protein (mg/L)	14.41	30.87	
Erythrocyte sedimentation rate (mm/H)	26	32.25	
Creatinine (μmol/L)	68.08	15.95	
Uric acid (μmol/L)	311.1	63.96	
Alanine aminotransferase (U/L)	33.43	43.03	
Aspartate transaminase (U/L)	29.43	16.14	
MI location			
Right coronary artery			3 (12%)
Left anterior descending branch			20 (80%)
Left main stem			1 (4%)
Circumflex branch			1 (4%)
Outcome*			
Complete remission			18 (72%)
Recrudesce			6 (24%)
Death			1 (4%)

* Outcome was evaluated for the 25 BS patients with MI and was classified as complete remission, Recrudesce (recurrent MI), and death.

Lesions were predominantly located in the left anterior descending (LAD) branch, accounting for 80% of cases, while other coronary territories, such as the right coronary artery, left main stem, and circumflex branch, were less frequently affected. This distribution suggests a predilection for specific coronary segments in BS‐related MI, which could have implications for treatment strategies and procedural planning.

Clinical outcomes were generally favorable, with 72% of patients achieving complete remission. However, 24% experienced recurrence, and 4% of patients succumbed to the condition, underscoring the variability in disease progression and response to treatment. These findings highlight the complex interplay between inflammatory markers, lesion distribution, and clinical outcomes in BS patients with MI. The results suggest that targeted management strategies addressing both systemic inflammation and localized coronary involvement could improve prognosis and reduce recurrence rates.

### 3.4 Differential Gene Expression and Pathway Analysis in BS and MI

**Figure 2:**
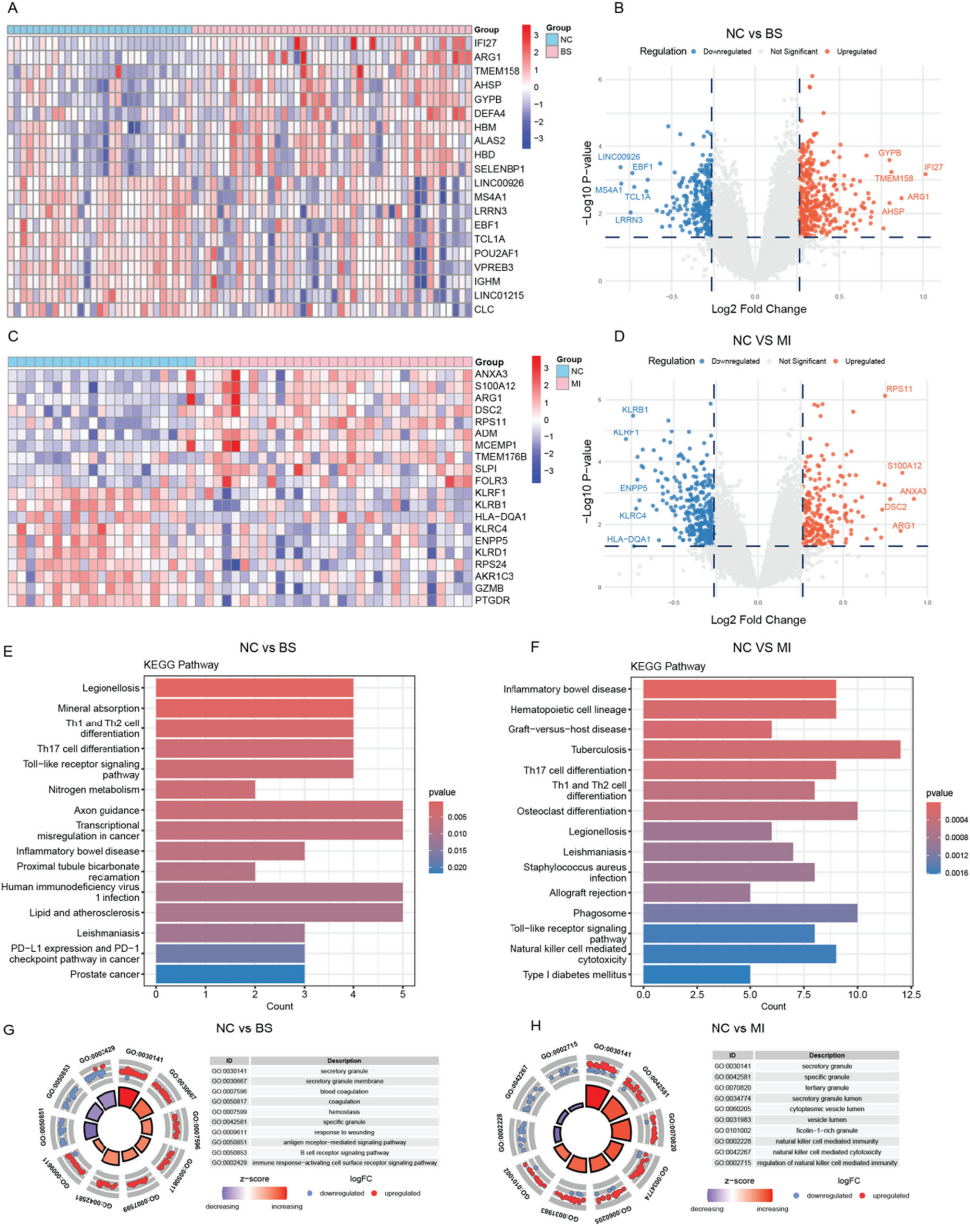
Differential gene expression and pathway analysis in BS and MI. (A) The heatmap illustrates the top 20 differentially expressed genes (10 upregulated and 10 downregulated) between the BS group and the control group. (B) The volcano plot displays all differentially expressed genes between the BS group and the control group, with upregulated genes in red, downregulated genes in blue, and nonsignificant genes in gray. (C) The heatmap illustrates the top 20 differentially expressed genes (10 upregulated and 10 downregulated) between the MI group and the control group. (D) The volcano plot displays all differentially expressed genes between the MI group and the control group, with upregulated genes in red, downregulated genes in blue, and nonsignificant genes in gray. (E and F) The bar plot shows the results of KEGG pathway enrichment analysis for the differentially expressed genes in the BS group and MI group. (G and H) The chord diagram illustrates the results of GO enrichment analysis for the differentially expressed genes in the BS group and MI group.

A total of 605 differentially expressed genes (DEGs) were identified in the comparison between the BS group and the control group, comprising 352 upregulated and 253 downregulated genes (Figure 2A–B). In the MI group, 523 DEGs were observed compared to controls, with 216 upregulated and 307 downregulated genes (Figure 2C–D).

KEGG pathway enrichment analysis revealed activation of immune‐related pathways in both BS and MI groups, albeit with distinct focal points. In BS patients, significant enrichment was observed in TH17 cell differentiation and Toll‐like receptor signaling, pathways central to autoimmune and inflammatory responses (Figure 2E). Conversely, MI patients exhibited substantial enrichment in hematopoietic cell lineage and natural killer cell‐mediated cytotoxicity, reflecting immune dysregulation with a focus on cellular defense and stress response mechanisms (Figure 2F).

GO analysis provided further distinction between the two conditions. BS was characterized by upregulation of immune activation and cytokine pathways, consistent with its autoimmune profile (Figure 2G). In contrast, MI demonstrated enrichment in pathways related to oxidative stress and extracellular matrix organization, indicative of vascular remodeling and inflammatory responses associated with cardiac injury (Figure 2H).

GSEA analysis highlighted shared hallmark pathways in both BS and MI, including upregulation in IL6/JAK/STAT3 signaling, coagulation, and complement activation, underscoring a common underlying pattern of immune dysregulation (Supporting Information Figure S1A,B). These findings suggest that, while BS and MI are driven by distinct pathophysiological mechanisms, they share overlapping immune and inflammatory responses. This shared immune activation highlights the potential for therapeutic strategies targeting immune modulation to benefit both conditions, particularly in addressing the immune imbalances that drive disease progression.

### 3.5 Identification of Significant Module Genes in BS and MI via WGCNA

**Figure 3:**
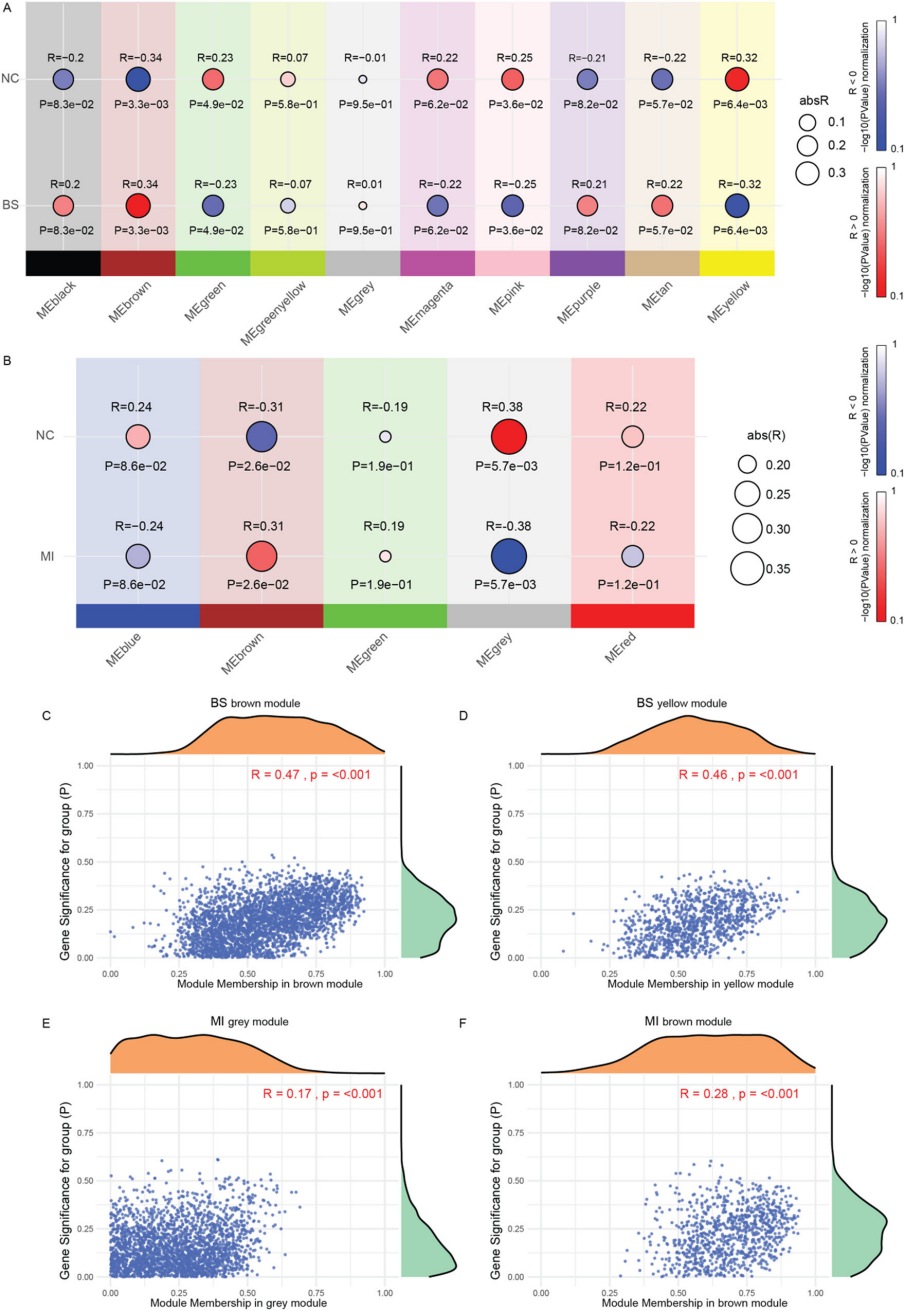
Identification of significant module genes in BS and MI via WGCNA. (A) The figure displays the correlation coefficients (R values) and *p*‐values for each module's association with the BS phenotype. (B) The figure displays the correlation coefficients (R values) and *p*‐values for each module's association with the MI phenotype. The size of the points corresponds to the absolute value of the R value, while the color indicates the direction of the correlation: blue for R < 0 and red for R > 0. (C and D) The scatter plots illustrate the correlation between gene significance and module membership in the two modules most strongly associated with the BS phenotype (brown and yellow modules). (E and F) The scatter plots illustrate the correlation between gene significance and module membership in the two modules most strongly associated with the MI phenotype (gray and brown modules).

Next, WGCNA was employed to identify significant gene modules associated with BS and MI. In the BS group, the brown and yellow modules demonstrated the strongest correlations, with correlation coefficients of R = 0.34 (P = 4.9 × 10⁻²) and R = 0.32 (P = 6.4 × 10⁻³), respectively, indicating that genes within these modules may play a critical role in BS pathogenesis (Figure 3A). In the MI group, the gray and brown modules showed notable correlations, with the gray module exhibiting a moderate negative correlation (R = −0.38, P = 5.7 × 10⁻³) and the brown module showing a positive correlation (R = 0.31, P = 2.6 × 10⁻²), suggesting that these modules may contain genes critical to MI‐related pathways (Figure 3B).

Gene significance scatter plots further validated the relevance of these modules. In BS, the brown and yellow modules displayed strong positive correlations with gene significance scores (R = 0.47 and R = 0.46, respectively, *p* < 0.001), reinforcing their importance in BS‐associated gene networks (Figure 3C–D). Similarly, in MI, the gray and brown modules showed significant correlations with gene significance (R = 0.17 for gray and R = 0.28 for brown, *p* < 0.001), highlighting their potential relevance in MI‐related pathways (Figure 3E–F). Supporting figures (Supporting Information Figure S2) illustrate the soft threshold selection and gene cluster tree, providing additional context for the module identification process.

### 3.6 Comprehensive Identification of Key Genes in BS and MI

**Figure 4:**
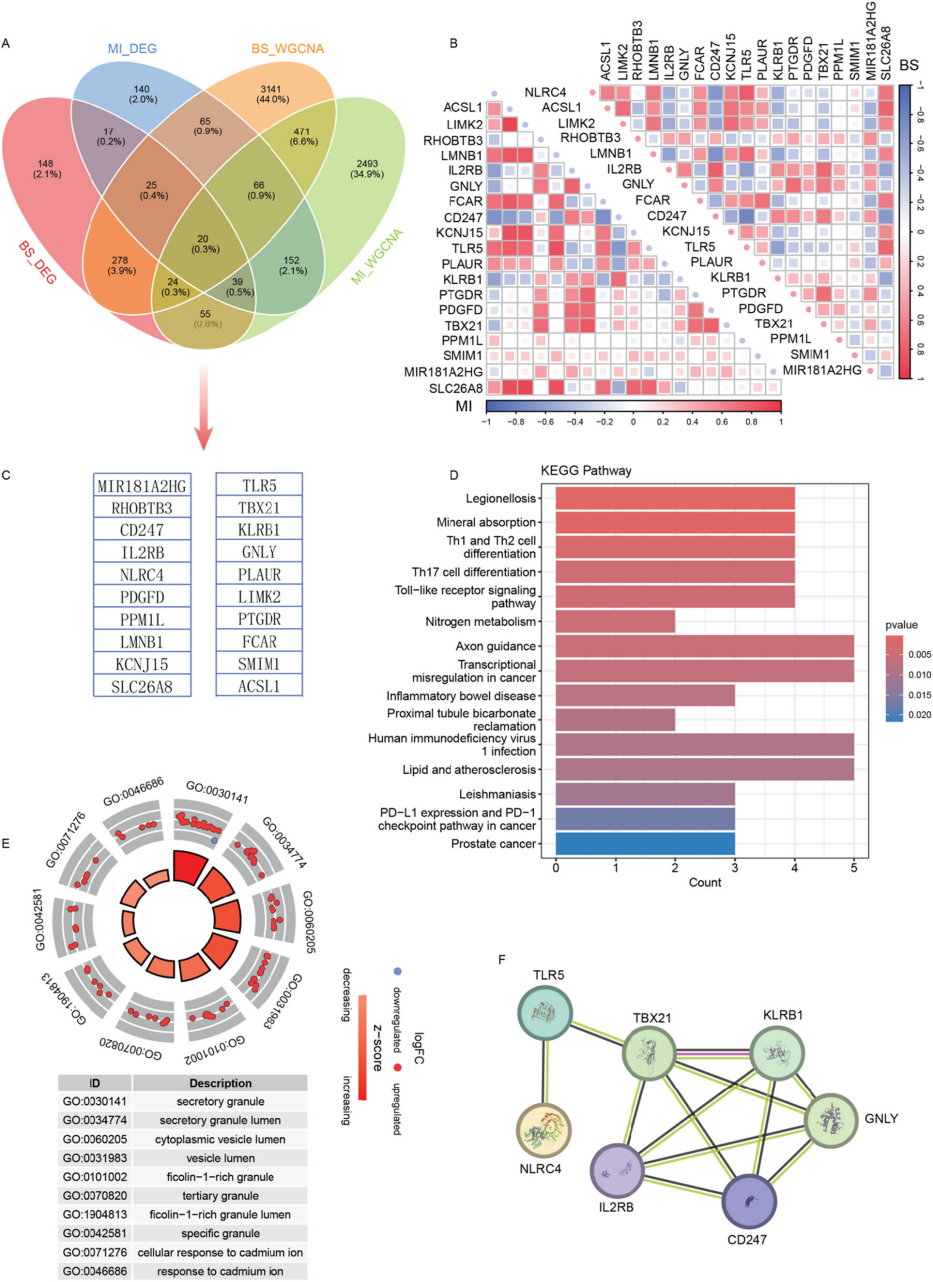
Comprehensive identification of key genes in BS and MI. (A) The Venn diagram reveals the intersection of DEGs from BS and MI datasets with WGCNA module genes. (B) The correlation heatmap demonstrates significant inter‐gene associations among the intersected genes in both BS and MI datasets. (C) A table lists the 20 key genes identified through the screening process. (D) The bar plot illustrates the results of KEGG pathway analysis conducted on the 20 key genes. (E) The chord diagram represents the results of GO functional analysis performed on the 20 key genes. (F) PPI analysis further identified seven critical genes from the 20 key genes.

To identify key genes associated with both BS and MI, we intersected the DEGs and WGCNA results from previous analyzes (Figure 4A), yielding 20 key related genes (Figure 4C). These genes were then analyzed separately in the BS and MI datasets to assess their associations (Figure 4B). KEGG pathway analysis revealed significant enrichment in immune and inflammatory pathways, including TH1 and TH2 cell differentiation, Toll‐like receptor signaling, and nitrogen metabolism. Pathways linked to chronic inflammation, such as lipid metabolism and atherosclerosis, were also prominently enriched (Figure 4D).

GO enrichment analysis highlighted cellular components involved in immune responses, including secretory granules and vesicle lumens, as well as responses to external stimuli, such as cadmium ion response. These findings suggest that the identified genes are implicated in immune modulation, inflammation, and cellular stress responses (Figure 4E).

Additionally, a protein‐protein interaction (PPI) network constructed from these genes identified TLR5, IL2RB, GNLY, CD247, KLRB1, NLRC4, and TBX21 as core genes (Figure 4F), suggesting their pivotal roles in the shared immune mechanisms underlying BS and MI.

### 3.7 Evaluation of ROC Curves and Nomogram Construction

**Figure 5:**
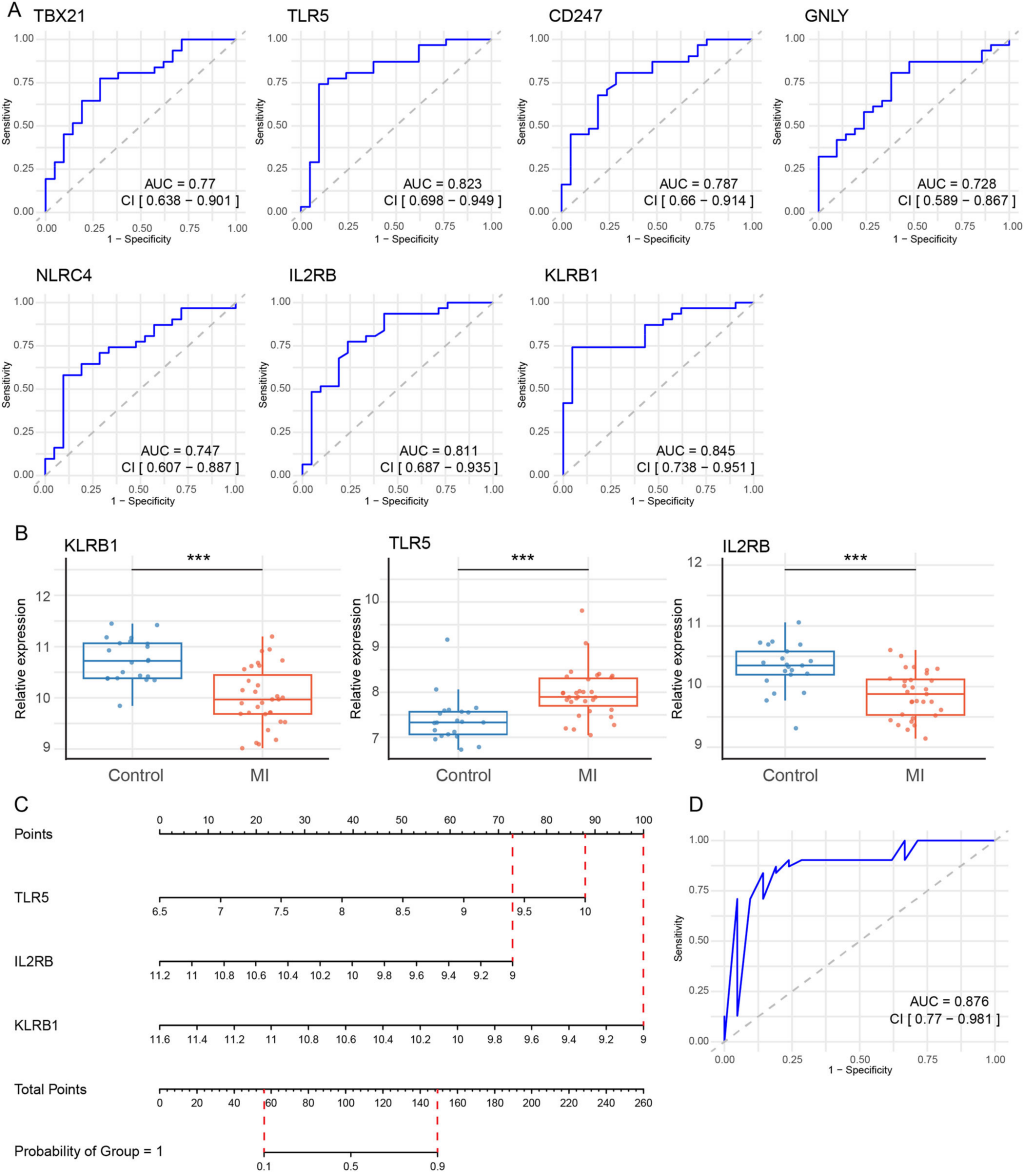
Evaluation of ROC curves and nomogram construction. (A) The diagnostic performance of the seven key genes was assessed using ROC curves. (B) Box plots display the expression levels of the three key genes (KLRB1, TLR5, and IL2RB) with AUC values > 0.7. (C) A nomogram model based on TLR5, IL2RB, and KLRB1 was constructed to predict MI risk. (D) The ROC curve for the nomogram model achieved an AUC of 0.876, further confirming its strong predictive performance for MI.

To further investigate key genes associated with BS and MI, we performed ROC curve analysis (Figure 5A), identifying three genes with AUC values exceeding 0.8: TLR5 (AUC = 0.823, 95% CI = 0.698–0.949), IL2RB (AUC = 0.811, 95% CI = 0.687–0.935), and KLRB1 (AUC = 0.845, 95% CI = 0.738–0.951). Expression analysis in the MI dataset revealed significant differential expression of these genes, with IL2RB upregulated and NLRC4 and KLRB1 downregulated in the MI group compared to controls (Figure 5B).

A nomogram incorporating TLR5, IL2RB, and KLRB1 was subsequently constructed (Figure 5C). ROC analysis of the nomogram demonstrated excellent diagnostic performance, with an AUC of 0.876 (95% CI = 0.770–0.981), indicating strong clinical diagnostic potential (Figure 5D). To validate its robustness, we conducted ROC curve analysis using the independent validation dataset GSE141512. The validation results were consistent with the initial findings, with AUC values for TLR5, IL2RB, and KLRB1 all exceeding 0.8 (Supporting Information Figure S3A). Expression patterns were similarly validated, showing low expression of IL2RB and KLRB1 and high expression of TLR5 in the MI group (Supporting Information Figure S3B). Notably, the nomogram achieved an AUC of 1.000 in the validation set, underscoring its exceptional diagnostic accuracy and clinical utility (Supporting Information Figure S3C,D).

## 4 Discussion

This study provides critical insights into the unique clinical features and underlying mechanisms of MI in BS, emphasizing the need for early vascular screening and intervention in BS patients to prevent severe cardiovascular events. Our findings reveal that MI patients have significantly longer disease durations compared to non‐MI patients, suggesting that chronic, unresolved inflammation and persistent vasculitis contribute to progressive vascular damage, thereby increasing the risk of cardiovascular complications. Although the age of onset did not significantly differ between the two groups, disease chronicity appears to play a pivotal role in the development of MI, with vascular involvement serving as a central mediator [30]. This strong association with disease duration reinforces the concept of vasculitis as a process of cumulative damage over time. Our finding is consistent with the landmark two‐decade outcome survey by Kural‐Seyahi et al., which demonstrated that morbidity in BS, including major organ damage, accrues over many years [7]. Our data suggest that while the age of MI onset may not differ significantly, it is the persistent, long‐term inflammatory burden and unresolved vascular injury that ultimately lower the threshold for catastrophic coronary events, distinguishing these patients from those with shorter disease histories.

Lifestyle factors, particularly smoking and alcohol consumption, were significantly more prevalent in the MI group, highlighting the role of traditional cardiovascular risk factors in BS [7]. Smoking, in particular, is known to exacerbate endothelial dysfunction and inflammation, which are central to the pathogenesis of BS‐associated vasculitis [7, 16]. This underscores the critical need for personalized health management strategies, including interventions targeting modifiable risk factors, to mitigate the likelihood of MI in this population. Furthermore, the significantly higher rates of vascular and arterial involvement in MI patients suggest that BS‐associated vasculitis accelerates endothelial injury, creating a pro‐thrombotic environment that predisposes individuals to thrombosis and arterial occlusion [11]. These findings advocate for early and comprehensive vascular screening and intervention in BS patients to prevent severe cardiovascular events.

Our study also highlights the systemic impact of BS on the cardiovascular system. Our observation that MI patients exhibit a high prevalence of concurrent major cardiac lesions—such as aortic aneurysms (28%) and significant valvular regurgitation—merits a direct comparison with previous literature. In their comprehensive review of 52 BS patients with various cardiac lesions, Geri et al. described a wide spectrum of involvement, including intracardiac thrombosis and endomyocardial fibrosis [9]. The pronounced rate of aortic structural damage specifically within our MI cohort suggests that the vasculitic process leading to coronary occlusion is part of a particularly aggressive systemic phenotype that concurrently targets the aortic root and valves. This supports the view that BS‐associated MI is often not an isolated coronary event but rather a sentinel marker for severe, widespread cardiovascular vasculopathy, underscoring the need for a holistic approach to patient care.

On a molecular level, this study provides novel insights into the pathophysiological mechanisms underlying MI in BS through the integration of gene expression analysis and weighted gene co‐expression network analysis (WGCNA). Our analysis identified critical overlapping inflammatory mechanisms, most notably the activation of the IL‐6/JAK/STAT3 signaling and complement pathways [31, 32], and pinpointed TLR5, IL2RB, and KLRB1 as key gene biomarkers with high diagnostic and predictive utility. The prominent role of the IL‐6/JAK/STAT3 pathway is particularly significant. As established by seminal work from Tanaka et al., IL‐6 is a pleiotropic cytokine that functions as a master regulator of the acute‐phase response and chronic inflammation [33]. Its role extends beyond a simple marker of inflammation; in cardiovascular disease, elevated IL‐6 actively promotes endothelial dysfunction, a pro‐coagulant state, and vascular smooth muscle proliferation, as detailed by Hansson GK [34]. Our finding thus provides a direct molecular bridge, linking the systemic immune dysregulation characteristic of BS to the established pathological pathways of vascular injury and thrombosis that precipitate MI.

Beyond these broad pathways, the identification of our specific gene biomarkers offers a more granular view of the potential pathogenesis. TLR5 is a key pattern recognition receptor of the innate immune system whose activation can trigger potent proinflammatory cascades implicated in other forms of vasculitis [35]. Meanwhile, IL2RB (CD122) and KLRB1 (CD161) are central to the function, activation, and homeostasis of lymphocyte populations, including regulatory T‐cells and cytotoxic NK cells, whose dysregulation is a known feature of BS pathology [36, 37]. The convergence of these findings—a major systemic inflammatory pathway combined with genes crucial to innate immunity and adaptive lymphocyte responses—strongly suggests that BS‐associated MI is driven by a specific nexus of immune activation. This not only provides a strong rationale for targeted anti‐inflammatory therapies but also underscores the potential of these biomarkers, integrated into a robust predictive nomogram as developed in our study, to pioneer a new approach for risk stratification in BS patients.

In line with recent literature, our findings reinforce the complexity of cardiovascular involvement in BS while offering novel molecular and clinical evidence that elucidates the unique characteristics of MI in this population [9, 38]. The emergence of precision medicine has created new opportunities for managing complex diseases like BS [39]. Leveraging the key genes and molecular pathways identified in this study, future research could focus on developing tailored diagnostic and therapeutic strategies, such as targeted anti‐inflammatory therapies, vascular protective agents, and comprehensive lifestyle interventions [33, 34].

We acknowledge several limitations in this study. The small size of our MI cohort (*n* = 25) limits the statistical power of clinical comparisons. Specifically, this limited sample size made it unfeasible to conduct subgroup analyzes comparing patients with and without traditional cardiovascular risk factors, making it difficult to be fully sure whether MI is causally related to BS itself or to these co‐existing risk factors. Furthermore, our bioinformatic analysis was based on public datasets, and our novel biomarkers currently lack experimental functional validation. Finally, the study's retrospective design meant potential confounders, such as specific medication regimens, were not systematically controlled. Future prospective, multi‐center studies that include functional validation are therefore required to confirm and expand upon our findings.

In conclusion, this study presents a comprehensive framework for understanding the clinical features, lifestyle influences, and molecular mechanisms of MI in BS. Future investigations should further explore the functional roles of the identified genes and validate their therapeutic potential as molecular targets. Moreover, integrating genetic, clinical, and molecular data into precision medicine models holds promise for optimizing the management of cardiovascular complications in BS, ultimately improving patient outcomes and quality of life.

## Author Contributions

**Li‐Yang Zhang:** writing – original draft, investigation, software, data curation, methodology. **Chun‐Hui She:** methodology, validation, supervision. **Hu Dan:** investigation, formal analysis. **Jun Zou:** visualization, formal analysis, data curation. **Jian‐Long Guan:** resources, writing – review and editing, funding acquisition, conceptualization, project administration.

## Ethics Statement

This study was approved by the Ethics Committee of Huadong Hospital Affiliated to Fudan University (Project no: 2018K031), all patients provided informed consent.

## Conflicts of Interest

The authors report no proprietary or commercial interest in any product mentioned or concept discussed in this article.

## Supporting information


**Supplementary Figure 1:** (A‐B) A bubble plot presents the results of GSEA analysis performed on DEGs from the BS group and MI group. **Supplementary Figure 2:** (A‐B) The selection of soft threshold power (β) for constructing scale‐free networks in the BS and MI groups is shown. **Supplementary Figure 3:** (A) ROC curves demonstrate the diagnostic capabilities of TBX21, IL2RB, and KLRB1 in the validation dataset. **Supplementary Table 1:** GEO datasets information.

## Data Availability

The data that support the findings of this study are available on request from the corresponding author.
