# Supplementary material for: Unraveling the Clinical and Molecular Landscape of Myocardial Infarction in Behçet's Syndrome: A Comprehensive Analysis
Source: Immun Inflamm Dis. 2025 Nov 27;13(11):e70303. doi: 10.1002/iid3.70303 (PMC12658460; doi:10.1002/iid3.70303)
Supplement: Supplementary file 1 — Supplementary Figure 1: (A‐B) A bubble plot presents the results of GSEA analysis performed on DEGs from the BS group and MI group. Supplementary Figure 2: (A‐B) The selection of soft threshold power (β) for constructing scale‐free networks in the BS and MI groups is shown. Supplementary Figure 3: (A) ROC curves demonstrate the diagnostic capabilities of TBX21, IL2RB, and KLRB1 in the validation dataset. Supplementary Table 1: GEO datasets information. [file IID3-13-e70303-s001.docx]

[Supplementary](https://authors.bmj.com/writing-and-formatting/formatting-your-paper/?_gl=1*7y8qrw*_gcl_au*MTA0Nzg5ODc5Mi4xNzMwNjM5NTk0*_ga*MjAzNDk2NTYyNS4xNjY3NzAxOTQw*_ga_EXTSVLH45V*MTczMzczMDA3OC4xNi4xLjE3MzM3MzA1MzAuMC4wLjE0NDYyMjU2MjM.*_fplc*NXV3ZE9rcWx3RTNESW9aUWMxOE4zbHlrdzhRaE1jN21lTVpCdE0xQ01aemN1V3NwMENvUUtQMWxES056UGxtTzV6ZDNtdVBpOEp6anJQNGhqc0lLWW11TkswUnRPMXUxUmtoNlRmT3lYQU9yVTVkVWdTOXZQTVd0ZzVQRHhBJTNEJTNE) table 1. GEO datasets information

| GSE series | Disease | Samples | Source types | Platform |
| --- | --- | --- | --- | --- |
| GSE17114 | BD | 15 BD patients and 14 normal controls | Peripheral blood | GPL570 |
| GSE209567 | BD | 29 BD patients and 15 normal controls | Peripheral blood | GPL570 |
| GSE48060 | MI | 31 MI patients and 21 normal controls | Peripheral blood | GPL570 |
| GSE141512 | MI | 6 MI patients and 6 normal controls | peripheral blood | GPL17586 |


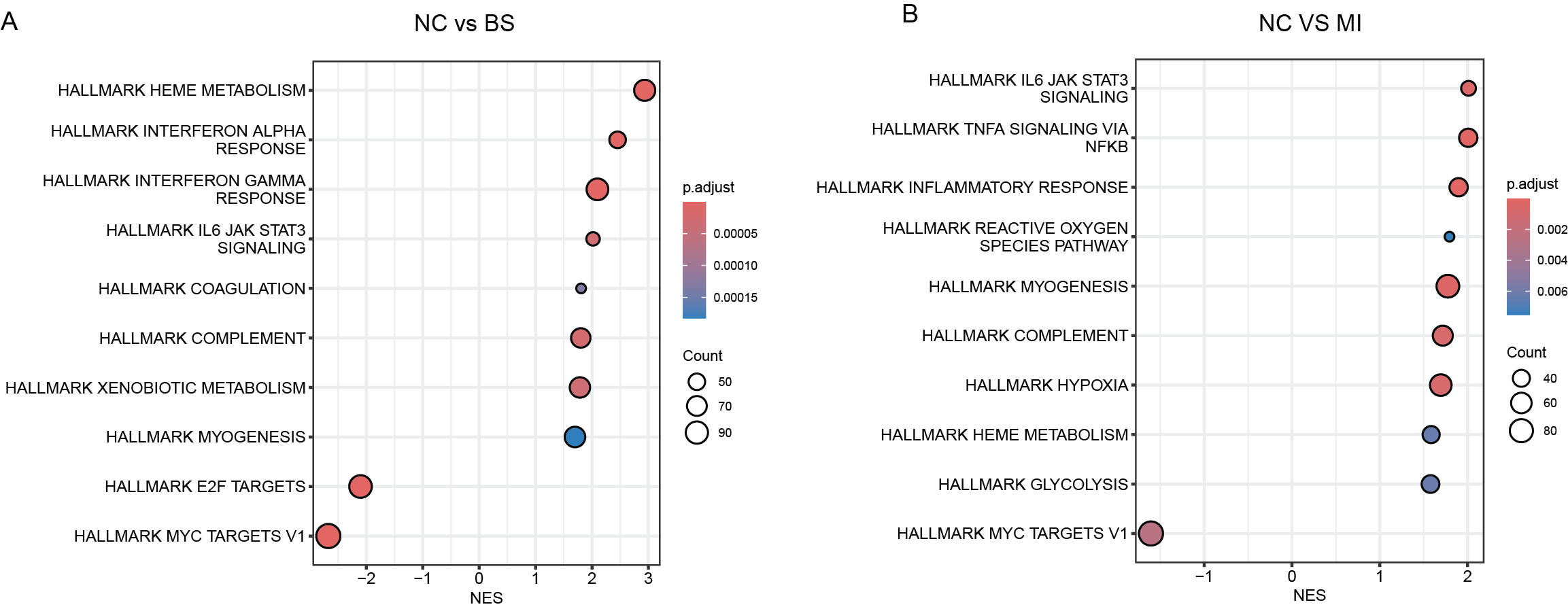


[Supplementary](https://authors.bmj.com/writing-and-formatting/formatting-your-paper/?_gl=1*7y8qrw*_gcl_au*MTA0Nzg5ODc5Mi4xNzMwNjM5NTk0*_ga*MjAzNDk2NTYyNS4xNjY3NzAxOTQw*_ga_EXTSVLH45V*MTczMzczMDA3OC4xNi4xLjE3MzM3MzA1MzAuMC4wLjE0NDYyMjU2MjM.*_fplc*NXV3ZE9rcWx3RTNESW9aUWMxOE4zbHlrdzhRaE1jN21lTVpCdE0xQ01aemN1V3NwMENvUUtQMWxES056UGxtTzV6ZDNtdVBpOEp6anJQNGhqc0lLWW11TkswUnRPMXUxUmtoNlRmT3lYQU9yVTVkVWdTOXZQTVd0ZzVQRHhBJTNEJTNE) Figure 1.

(A-B) A bubble plot presents the results of GSEA analysis performed on DEGs from the BS group and MI group.


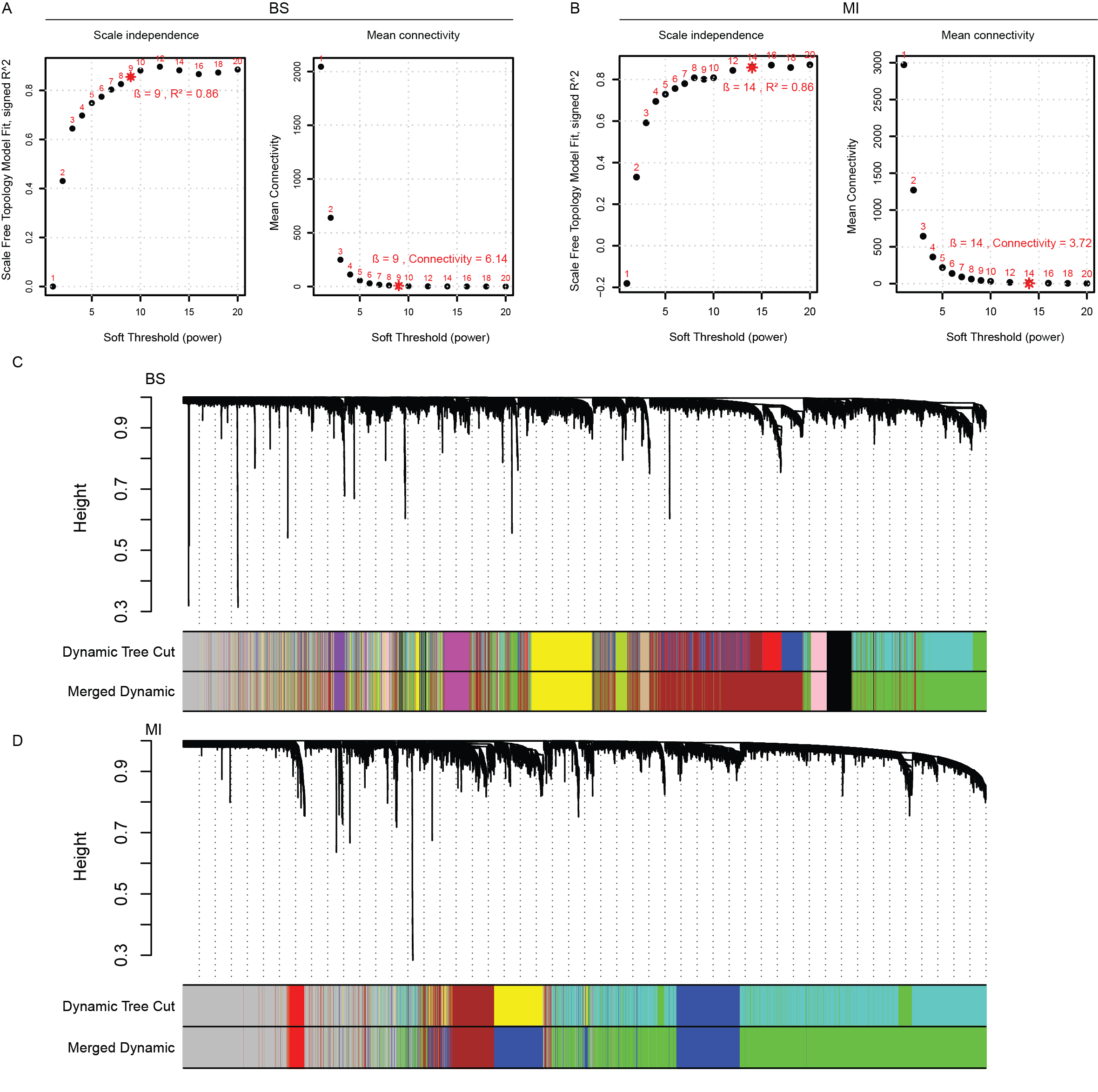


[Supplementary](https://authors.bmj.com/writing-and-formatting/formatting-your-paper/?_gl=1*7y8qrw*_gcl_au*MTA0Nzg5ODc5Mi4xNzMwNjM5NTk0*_ga*MjAzNDk2NTYyNS4xNjY3NzAxOTQw*_ga_EXTSVLH45V*MTczMzczMDA3OC4xNi4xLjE3MzM3MzA1MzAuMC4wLjE0NDYyMjU2MjM.*_fplc*NXV3ZE9rcWx3RTNESW9aUWMxOE4zbHlrdzhRaE1jN21lTVpCdE0xQ01aemN1V3NwMENvUUtQMWxES056UGxtTzV6ZDNtdVBpOEp6anJQNGhqc0lLWW11TkswUnRPMXUxUmtoNlRmT3lYQU9yVTVkVWdTOXZQTVd0ZzVQRHhBJTNEJTNE) Figure 2.

(A-B) The selection of soft threshold power (β) for constructing scale-free networks in the BS and MI groups is shown. (C-D) Gene module dendrograms for the BS and MI conditions, respectively, were generated, identifying distinct gene clusters.


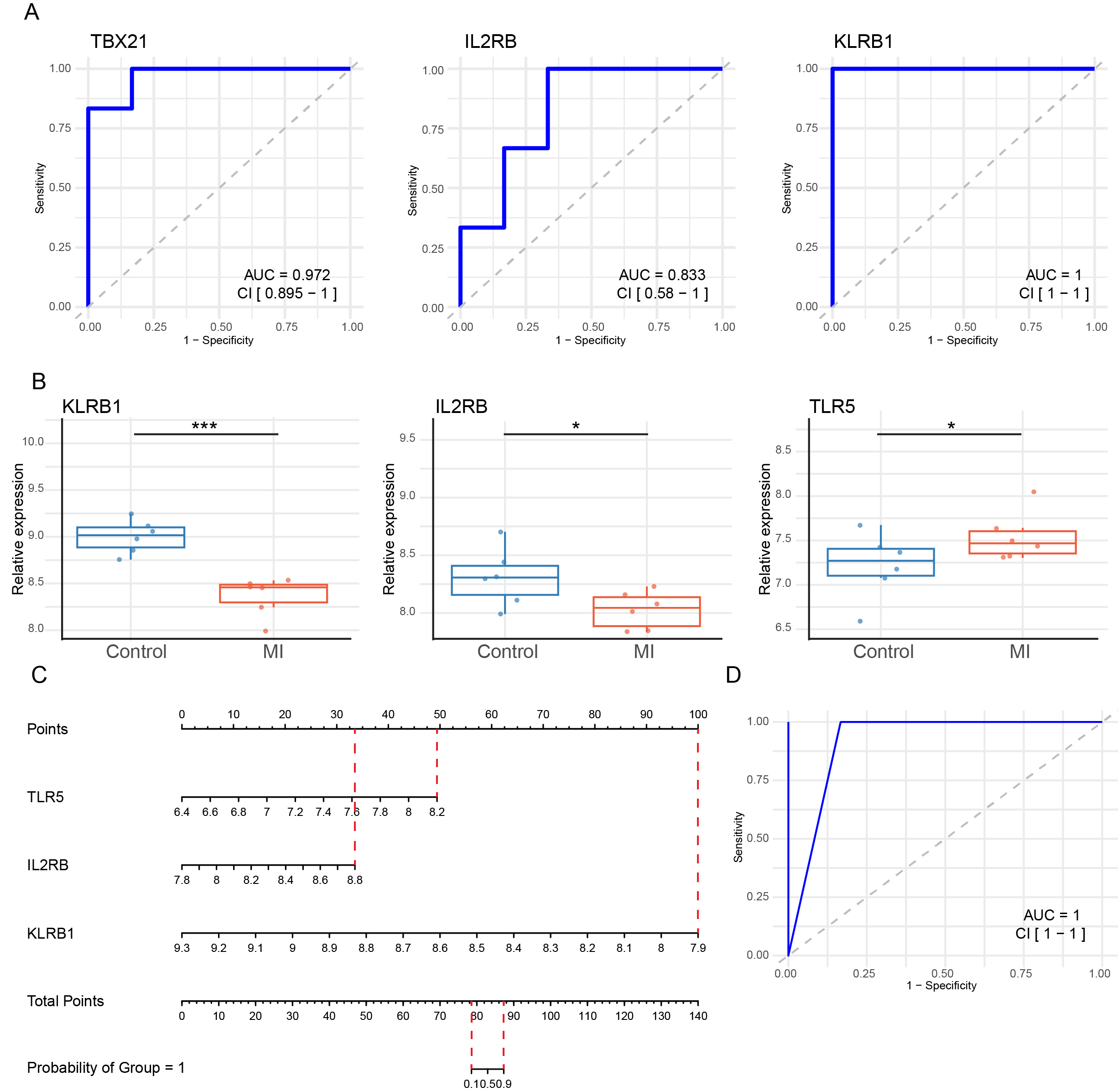


[Supplementary](https://authors.bmj.com/writing-and-formatting/formatting-your-paper/?_gl=1*7y8qrw*_gcl_au*MTA0Nzg5ODc5Mi4xNzMwNjM5NTk0*_ga*MjAzNDk2NTYyNS4xNjY3NzAxOTQw*_ga_EXTSVLH45V*MTczMzczMDA3OC4xNi4xLjE3MzM3MzA1MzAuMC4wLjE0NDYyMjU2MjM.*_fplc*NXV3ZE9rcWx3RTNESW9aUWMxOE4zbHlrdzhRaE1jN21lTVpCdE0xQ01aemN1V3NwMENvUUtQMWxES056UGxtTzV6ZDNtdVBpOEp6anJQNGhqc0lLWW11TkswUnRPMXUxUmtoNlRmT3lYQU9yVTVkVWdTOXZQTVd0ZzVQRHhBJTNEJTNE) Figure 3.

(A) ROC curves demonstrate the diagnostic capabilities of TBX21, IL2RB, and KLRB1 in the validation dataset. (B) Box plots illustrate the expression levels of TBX21, IL2RB, and KLRB1 in the validation dataset. (C) A nomogram model incorporating TLR5, IL2RB, and KLRB1 was developed in the validation dataset for MI risk prediction. (D) The ROC curve of the nomogram model achieved an AUC of 1 in the validation dataset, indicating excellent predictive performance for MI.
